# A Review of the Use of Native and Engineered Probiotics for Colorectal Cancer Therapy

**DOI:** 10.3390/ijms25073896

**Published:** 2024-03-31

**Authors:** Huawen Han, Yifan Zhang, Haibo Tang, Tuoyu Zhou, Aman Khan

**Affiliations:** 1 State Key Laboratory of Grassland Agro-Ecosystems, College of Pastoral Agriculture Science and Technology, Lanzhou University, Lanzhou 730000, China; 2 College of Medical, Veterinary and Life Sciences, University of Glasgow, Glasgow G12 8QQ, UK; zyf03111998@163.com; 3 Ministry of Education Key Laboratory of Cell Activities and Stress Adaptations, School of Life Science, Lanzhou University, Lanzhou 730000, China; tanghb20@lzu.edu.cn (H.T.); zhouty19@lzu.edu.cn (T.Z.); 4 College of Life Sciences, Northeast Forestry University, Harbin 150040, China

**Keywords:** colorectal cancer, synthetic biology, probiotics and engineered strain

## Abstract

Colorectal cancer (CRC) is a serious global health concern, and researchers have been investigating different strategies to prevent, treat, or support conventional therapies for CRC. This review article comprehensively covers CRC therapy involving wild-type bacteria, including probiotics and oncolytic bacteria as well as genetically modified bacteria. Given the close relationship between CRC and the gut microbiota, it is crucial to compile and present a comprehensive overview of bacterial therapies used in the context of colorectal cancer. It is evident that the use of native and engineered probiotics for colorectal cancer therapy necessitates research focused on enhancing the therapeutic properties of probiotic strains.. Genetically engineered probiotics might be designed to produce particular molecules or to target cancer cells more effectively and cure CRC patients.

## 1. Introduction

Colorectal cancer (CRC) is the most commonly diagnosed cancer and the leading cause of death worldwide [1]. Mutations in specific genes, including oncogenes, tumor suppressor genes, and genes associated with DNA repair mechanisms, are responsible for the development of CRC [2]. At present, the choice of CRC treatment measurement is generally based on tumor-related characteristics and patient information [2]. Chemotherapeutic drugs (5-Fu and oxaliplatin) and targeted drugs (cetuximab and bevacizumab), as the first choice for treatment for patients with advanced CRC, can significantly improve patient survival. These drugs have systemic administration-related toxic side effects in the treatment of the CRC. and may lead to a weaker treatment response due to the heterogeneity of CRC [3,4]. Meanwhile, the relative lack of new specific biomarkers also hinders the development of more effective targeted drugs [5]. Therefore, it is critical to develop effective and alternative methods for CRC treatment.

The gut microbiota is closely related to the host’s health. CRC pathogenesis is often accompanied by intestinal microbial dysbiosis. Increased abundances of multiple gut bacteria, including *Fusobacterium nucleatum* and *Peptostreptococcus anaerobius*, promotes colorectal carcinogenesis [6,7]. Probiotic bacterial strains can restore gut microbial homeostasis via the modulation of gut microbiota [8,9]. The prevention or treatment of CRC using probiotics has become a potential strategy [8]. Similarly, the use of oncolytic bacteria such as *Salmonella typhimurium* have also been researched as a CRC treatment strategy [10,11,12]. This strain induces tumor regression through various mechanisms, including the direct induction of apoptosis; the depletion of nutrients and oxygen; and the activation of the tumor-specific immune response [13]. However, wild bacteria have weak therapeutic ability and uncontrolled physiological behavior. The characteristics of simple metabolic activity and single therapeutic function make it difficult to cope with malignancy efficiently [14]. Meanwhile, viable microorganisms may cause unpredictable harm to the host, including infections, inflammatory effects, and carcinogenesis [15,16]. Therefore, wild-type bacteria are still far from being an ideal tumor therapeutic agent.

With great advancements in synthetic biology and biomedical engineering, the therapeutic benefits of wild-type bacteria have been enhanced via directional genetic modifications. The anti-tumor ability of bacteria is further improved. Genetically modified bacteria can be employed as live vectors to deliver various therapeutic payloads, including cytotoxic proteins, immunoregulatory factors, prodrug-converting enzymes, angiogenesis regulation proteins, RNA interference (RNAi) molecules, and immunoregulatory factors [17,18]. Meanwhile, the development of bacterial-component-based strategies has also been applied to CRC therapy, such as outer engineered membrane vesicles (OMVs), which are nanosized vesicles with excellent tumor penetrability [19]. In addition, the targeting of genetically modified bacteria is further optimized for precise intervention at tumor lesion sites. One strategy involves enhancing the capacity of bacteria to bind to tumor cells. For instance, bacteria were genetically altered to produce the Histone-like protein A (HlpA), known for its strong affinity to the surfaces of colorectal cancer (CRC) cells, thereby enhancing targeting efficacy [20]. Another strategy involves the precise control of therapeutic payload expression and release. One typical example is the integration of inducible promoters into the gene expression system for the control of target gene expression at a specific location, which can effectively avoid the off-target delivery of toxic drugs [21].

Unlike other therapeutics, bacteria-mediated tumor therapy is not directly affected by the “genetic makeup” of the tumor [22], which makes it a unique tumor treatment strategy. Although many exciting research findings related to this aspect have been summarized and discussed in previous reviews, a noticeable feature remaining today is that many reviews focus on various types of cancer [23,24]. This review article focuses on CRC therapy using wild-type bacteria (including probiotics and oncolytic bacteria) and genetically modified bacteria. Given the close association between CRC and the gut microbiota, it is necessary to summarize the application of bacterial therapies in CRC. Thus, in contrast to other reviews, we seek to fill this gap and carefully summarize the recent reports on bacteria and the bacterial delivery of therapeutic payloads for the treatment of CRC. This is followed by a discussion of different strategies to improve bacterial tumor-targeting ability [22,25]. The ultimate goal is to provide a reference for the development of more efficient and safer genetically engineered bacteria for CRC treatment in clinics.

## 2. Native Bacterial Approach for Colorectal Cancer (CRC) Treatment

**Figure 1:**
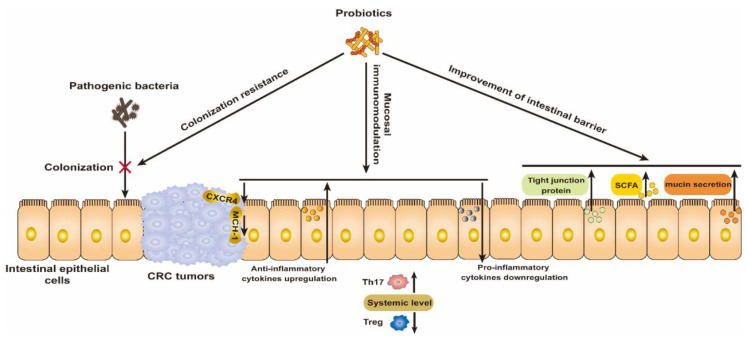
Mechanism of wide-type probiotics for the prevention and treatment of CRC via modulating the colonization resistance of pathogenic bacteria, mucosal immunomodulation, and improving the intestinal barrier.

The success of the Bacillus Calmette–Guérin (BCG) vaccine for the treatment of bladder cancer highlights the great potential of bacteria for cancer treatment [26]. Probiotics in particular are recognized to play a prominent role in the prevention and treatment of CRC by modulating the gut microbiota. Thus, we briefly summarize the mechanism by which probiotics resist CRC and their preliminary clinical benefits (Figure 1).

### 2.1. Colonization Resistance for Pathogenic Bacteria

The occurrence of CRC is closely related to environmental factors, diet, and gut microbiome composition [27]. The development of CRC was usually accompanied by an increase in the abundance of intestinal pathogenic bacteria in a multi-cohort analysis of 526 CRC patients’ samples; these bacteria included *Fusobacterium nucleatum*, *Peptostreptococcus anaerobius*, *Prevotella intermedia*, *Alistipes finegoldii*, *Thermanaerovibrio acidaminovorans*, and enterotoxigenic *Bacteroides fragilis* [28], which contribute to tumor proliferation by promoting inflammation, causing DNA damage, etc. Another pathological epidemiology database of 1069 rectal and colon cancer cases revealed that the amount of *F. nucleatum* DNA in colorectal cancer tissue is associated with shorter survival [29]; thus it can serve as a prognostic biomarker. Furthermore, most probiotics with a protective effect against CRC have been exhausted, including *Lachnospiraceae* species, *Bifidobacterium animalis*, and *Streptococcus thermophilus*, and are found to be depleted in CRC patients [30]. Thus, probiotic intervention can resolve the dysbiosis of the gut ecosystem. Through colonization resistance, probiotic strains were able to reduce the colonization of pathogens such as *Clostridium difficile,* which has been recognized as a cancer-stimulating bacterium, *in Apc^Min/+^ mice* [31]. In addition, metabolites produced by probiotics such as lactic acid and acetic acid make an acidic lumen environment to inhibit the growth of pathogenic bacteria [8]. Metabolite bacteriocins can also directly kill pathogens to maintain intestinal microbial health [32]. Thus, these properties of probiotics help restore the intestinal dysbiosis associated with CRC.

### 2.2. Mucosal Immunomodulation

The benefits of probiotics on the gut are inseparable from their intestinal immunomodulatory effects. Inflammatory cytokines were involved in colon carcinogenesis in 17 cases of human colon adenocarcinomas [33]. Dysbiosis of the intestine induces the secretion of pro-inflammatory cytokines and nitric oxide, which results in the occurrence of inflammation and CRC. Probiotics contribute to the reconstruction of the intestinal ecological balance and regulate the secretion of cytokines to restore intestinal homeostasis [22,25]. For example, a synbiotic combination of the probiotic *Lactobacillus gasseri* 505 and a prebiotic *Cudrania tricuspidata* leaf extract exerted a cancer-protective effect by downregulating pro-inflammatory cytokines in an azoxymethane (AOM)/dextran sodium sulfate (DSS)-induced CRC mouse model [34]. In addition, some studies show that probiotics improve antitumor immunity at a systemic level by enhancing and reducing the expression of T helper 17 (Th17) and regulatory T (Treg) cells, respectively, as well as inhibiting the expression of tumor C-X-C chemokine receptor type 4 (CXCR4) and major histocompatibility complex class I (MHC-1) [35]. *Lactobacillus plantarum* from traditional fermented milk products was reported to show anti-CRC activities in a CT26 subcutaneous-tumor mouse model [36]. The oral administration of *L. plantarum* suppressed tumor growth by increasing CD8^+^ and natural killer (NK) cell infiltration into the tumor and promoting Th1-type CD4^+^ T differentiation and the upregulation of interferon-γ (IFN-γ) levels [37]. Another study involved in checkpoint blockade immunotherapy revealed that the combinative administration of *L. acidophilus* lysates and CTLA-4 mAb cytotoxic T-lymphocyte-associated protein 4 (CTLA-4) blocking antibodies inhibited colon carcinogenesis in AOM/DSS syngeneic BALB/c mice [37]. The treatment outcome was associated with increased numbers of CD8^+^ T cells and effector memory T cells and decreased numbers of Treg and M2 macrophages [37].

### 2.3. Improvement of Intestinal Barrier

Intestinal epithelial cells protect the internal environment from pathogenic bacteria and toxic substances. Tight junction proteins, as components of the intestinal barrier, are disrupted by intestinal pathogens, and their toxins, CRC metastasis, and tumor invasion are enhanced [38]. Tight junction integral protein claudin-3 serves as a conjoint rheostat for regulating Stat-3 and Wnt/β-catenin activation to prevent CRC malignancy, which makes it an important therapeutic target for CRC suppression [39]. The probiotic Escherichia coli Nissle 1917 (EcN), along with its released active factors and outer membrane vesicles (OMVs), facilitated the upregulation of tight junction proteins such as ZO-1 and claudin-14, thereby enhancing the integrity of the intestinal barrier. [40]. Short-chain fatty acids (SCFAs), including butyrate, propionate, and acetate, are secreted by probiotics and help to improve intestinal barrier function. In an in vitro study, SCFAs reduced metabolic stress and inhibited ethanol damage to tight junction proteins by activating AMP-activated protein kinase (AMPK) in caco-2 cells [41]. In an AOM/DSS model of colitis, propionate enhanced barrier function as well as reduced inflammation and oxidative stress caused by dextran sodium sulfate (DSS) [42]. In addition, mucin secreted by goblet cells in the intestinal epithelium is indispensable in strengthening the defenses of the intestinal barrier [43]. Probiotics can enhance the production of intestinal mucin. For example, *L. rhamnosus* CNCM I-3690 resisted damage to the intestinal barrier caused by inflammation via the regulation of the goblet cells and mucus layer of the intestinal tract in dinitrobenzene sulfonic acid (DNBS)-induced C57BL/6 mice [44]. Probiotic-mediated mucin expression also may be a strategy to colonize beneficial microorganisms into the host’s gut.

### 2.4. The Effect of Probiotics on CRC in Terms of Clinical Research

Promising data in preclinical models and the results of clinical research on probiotics have been reported. Two earlier randomized controlled trials evaluated the effects of probiotics for the prevention of CRC [45,46]. The results of a four-year clinical trial indicated that *L. casei* prevented the atypia of colorectal tumors, whereas the administration of dietary fiber and *L. casei* combinations did not inhibit the development of new tumors [45]. A 12-week randomized, double-blind, placebo-controlled trial showed that a dietary symbiotic comprising oligofructose-enriched inulin, *L. rhamnosus* GG, and *Bifidobacterium lactis* Bb12 suppressed intestinal inflammation and reduced genotoxin exposure [46]. Recently, growing attention has been paid to the use of probiotics in CRC patients to alleviate conventional treatment-induced side effects. For example, a 4-week randomized, double-blind, placebo-controlled trial explored the effects of probiotics containing six viable microorganisms of *Lactobacillus* and *Bifidobacteria* strains on postsurgical complications in patients with colorectal cancer. The results suggested that they could modulate the intestinal microenvironment and result in a decrease in pro-inflammatory cytokines [47]. Another randomized, double-blind, placebo-controlled study involving 73 CRC patients analyzed the effect of preoperative symbiotic (Simbioflora^®^) or placebo (maltodextrin) administration in CRC patients and found that preoperative symbiotic administration for 7 days prevented inflammation and reduced morbidity, length of hospital stay, and antibiotic use in CRC patients Table 1 [48]. Additionally, one study also reported that the use of probiotics in the perioperative period was effective in reducing the rate of infection after CRC surgery [49]. However, heterogeneity present in trials with small sample sizes requires that conclusions be proved by larger randomized controlled trials. Meanwhile, it should be investigated whether the clinical benefits of these short-term studies translate into improved long-term outcomes.

Using wild-type probiotic bacteria to target and kill tumors is a promising strategy [50]. However, the therapeutic capacity of each bacterium is single and limited, and it is difficult to deal with complex malignancies. Genetic engineering and synthetic biology techniques can be used to modify bacteria to improve their beneficial properties and, most importantly, their therapeutic efficacy and targeting capabilities [51]. Balancing the bacterial dosage required for tumor destruction with toxicity or adverse effects on the host is a significant challenge. Additionally, the pathogenicity of these bacteria remains a concern [36]. Despite attempts to reduce the pathogenicity of these bacteria through gene deletion, complete success has not yet been achieved [52]. Adverse effects and mortality rates have been reported when using these bacteria for treatment [53,54]. Finally, these bacteria are not entirely capable of specifically identifying and attacking tumor cells. Some bacteria may aggregate in normal tissues and the bloodstream, leading to systemic infections or other adverse effects. 

## 3. Genetically Modified-Bacteria for Colorectal Cancer (CRC) Treatment

Although naive bacteria have insufficiently potent anti-tumor properties, the excellent heterologous protein production capacity of these bacteria has made it possible to improve their effectiveness as anti-CRC agents. Here, we describe in detail the use of different strategies to modify wild-type bacteria to deliver anti-cancer molecules.

### 3.1. Engineered Bacteria Treat CRC via Therapeutic Protein Delivery

#### 3.1.1. Cytotoxic Proteins

**Figure 2:**
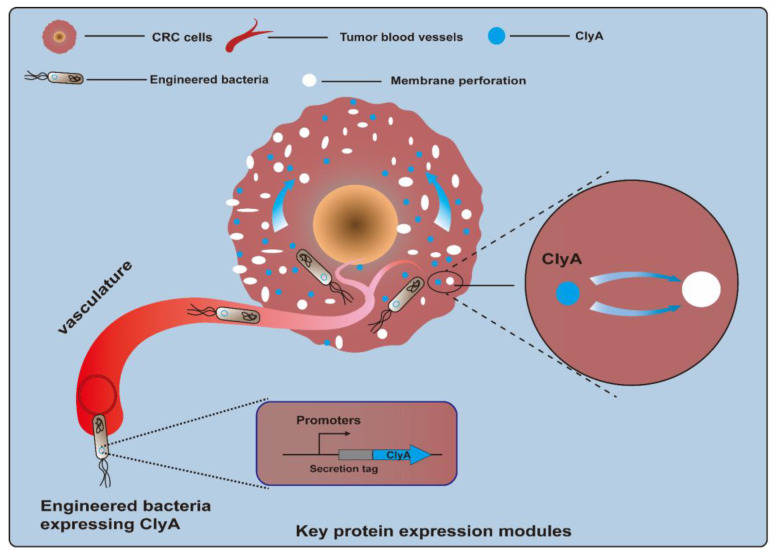
Engineered bacteria harboring cytolysin A protein (ClyA, a pore-forming cytotoxin) can act on tumor cells by forming transmembrane pores and inducing cell apoptosis.

Cytotoxic proteins, a kind of biological toxin, are produced by a variety of different organisms and are poisonous to animal cells. Killing CRC cells via cytotoxin is a feasible method of CRC treatment. However, because of a lack of selectivity for protein toxins, normal cells may be accidentally injured. Bacteria can specifically colonize tumor sites, which provides an opportunity for toxic proteins to directly contact tumor cells and exert their toxic effects [55,56]. The cytolysin A (ClyA) protein, also referred to as hemolysin E (HlyE), is a pore-forming cytotoxin expressed by *E. coli* and enterobacteria (Figure 2). It can cause the formation of transmembrane pores on mammalian cells and macrophages and induce cell apoptosis [57,58]. A study described that engineered *E. coli* K-12-expressing ClyA could significantly decrease tumor growth rates, and a combination of therapy and radiation was effective in suppressing tumor growth and metastasis in a CT26 mouse model [59]. Recently, a biohybrid therapeutic platform based on *E. coli* K-12 was developed to express ClyA. This treatment, in combination with photothermal therapy, significantly inhibited tumor growth for BALB/c mice bearing CT26 tumors [60]. In another example, engineered *E. coli* K-12-secreting ClyA arrested tumor growth and impeded tumor metastasis by triggering thrombosis and killing tumor cells in CT26 tumor tissues [61]. Another approach involved using an engineered attenuated *S. typhimurium* ΔppGpp strain as a bacteria chassis to express ClyA. This strategy could significantly suppress both primary and metastatic tumors and prolong survival in CT26 tumor-bearing mice [62,63]. Notably, a recent study employed an *E. coli* Nissle 1917 (EcN) strain as a chassis to deliver the protein toxin HlyE. The engineered EcN strain was designed to enable the expression of HlyE in a temporally controlled manner under the control of the araBAD promoter. As a result, this engineered strain preferred to be colonized in tumor tissues and induced tumor regression in mice xenografted with either human CRC cells [64]. Other types of cytotoxic proteins have also been tested for their anti-CRC efficacy. TGFα-PE38 is a cytotoxic protein comprising transforming growth factor alpha (TGFα), an EGFR ligand, and a modified *Pseudomonas* exotoxin A (PE38), a potent cytotoxic protein. Attenuated *S. typhimurium* armed with a recombinant TGFα-PE38 fusion protein led to notable retardation in solid tumor growth observed in BALB/c mice bearing CT26 tumors, C57BL/6 mice bearing MC38 tumors, and nude mice bearing SW620 tumors [65].

#### 3.1.2. Prodrug-Converting Enzymes

The targeted delivery of prodrug-converting enzymes with engineered bacteria is a therapeutic strategy for converting prodrugs into cytotoxic products at the tumor site. It aims to yield effective concentrations of anticancer drugs in the tumor microenvironment (TME) along with minimized adverse effects on organisms. Cytosine deaminase (CD), produced by bacteria and fungi, is a kind of representative prodrug-converting enzyme. CD can convert 5-fluorocytosine (5-FC), a non-toxic prodrug, into 5-fluorouracil (5-FU), a toxic chemotherapeutic agent [66]. A study was designed to develop an engineered attenuated strain of *S. typhimurium* expressing CD from *E. coli*. In an MC38 tumor-bearing C57BL/6 mouse model, this engineer accumulated in tumors at levels 1000-fold higher than those in normal tissues after a single intravenous bolus administration. High levels of 5-FU were detected in tumor tissues when MC38 mice were treated with this engineered strain and 5-FC. In addition, an anti-tumor effect was observed in MC38 mice treated with both engineered bacteria alone and engineered bacteria in combination with 5-FC but not in mice treated with 5-FC alone [67]. Interestingly, wild-type probiotic EcN is inherently capable of activating several prodrugs including CB1954, 5-FC, AQ4N, and Fludarabine phosphate, and resisting their toxicity. The combination administration of EcN and prodrugs showed a significant antitumor effect in BALB/c mice bearing CT26 tumors [68]. Even though this activation mechanism of EcN has not been elucidated, it serves as a natural enzyme pool that will facilitate the development of more prodrug-converting enzymes.

Given the higher nutrient demand for amino acids in cancer cells, it is probably an efficient cancer therapeutic strategy to employ bacteria to deliver specific amino-acid-depletion enzymes for the deprivation of essential amino acids in the TME. Asparaginase (L-ASNase) of *E. coli* origin is an anti-cancer protein for acute lymphoblastic leukemia [69]. It converts asparagine into aspartate and glutamine into glutamate at a low level. Some cancer cell types have no or little asparagine synthetase expression, so they cannot convert aspartate back into asparagine for further use [70]. In this case, cancer cells will suffer from asparagine starvation and subsequently experience apoptosis. Kim et al. engineered attenuated *S. typhimurium* to produce L-ASNase to counter tumors. This strain expressed L-ASNase under the control of the inducible araBAD promoter system for the control of gene-selective expression within solid tumors. As a result, the L-ASNase expressed and secreted by *S. typhimurium* induced apoptotic cancer cells in vitro, and this engineered strain significantly promoted tumor regression and increased survival in an MC38 mouse model [71].

**Figure 3:**
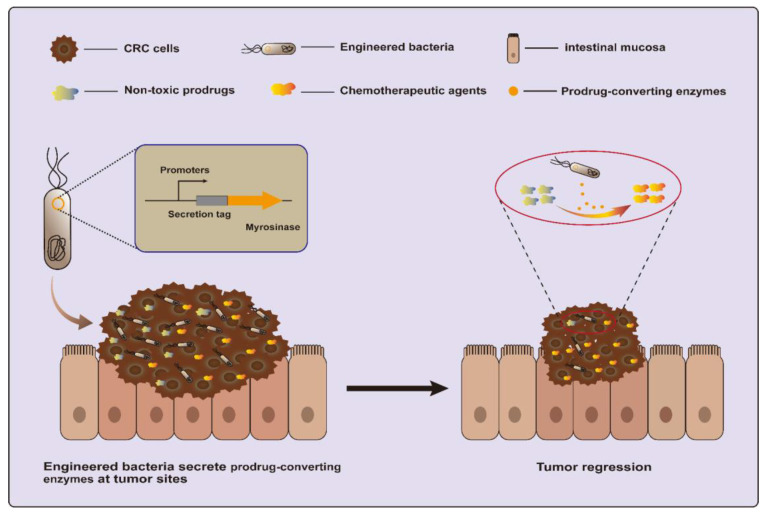
Engineered bacteria harboring myrosinase can convert dietary glucosinolates into anti-cancer molecule sulforaphane for CRC treatment.

Another strategy is to utilize a dietary prebiotic as a prodrug and convert it into anticancer compounds using specific enzymes. One recent study engineered EcN to secrete myrosinase, an enzyme that converts dietary glucosinolates, naturally occurring components of cruciferous vegetables, into the anti-cancer molecule sulforaphane (Figure 3). This strategy succeeded in killing over 95% of human and murine CRC cell lines in vitro and reducing over 75% of tumors in azoxymethane (AOM) and dextran sodium sulfate salt (DSS)-induced CRC mice [20,72].

#### 3.1.3. Angiogenesis Regulation Proteins

**Figure 4:**
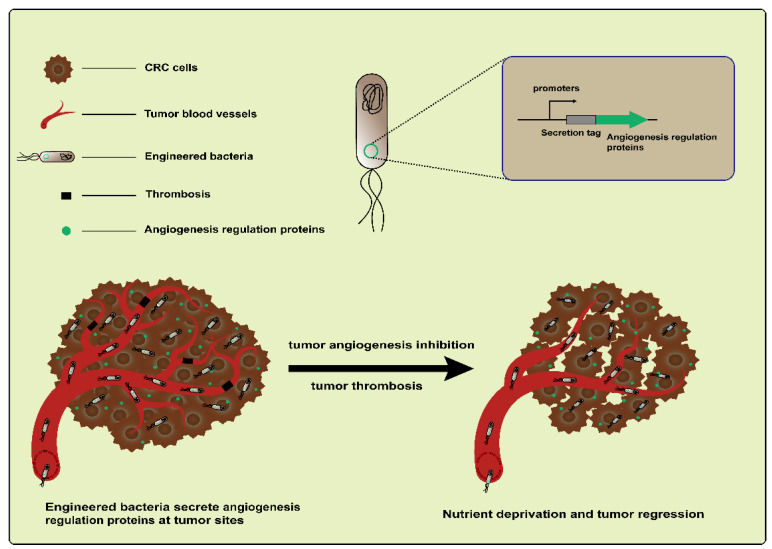
Engineered bacteria harboring angiogenesis regulation proteins can inhibit the suppression of tumor angiogenesis via targeting and destroying blood vessels.

Tumor angiogenesis is an important feature in CRC development [73]. Based on targeting tumor blood vessels to treat CRC using engineered bacteria, both antiangiogenesis and vascular destruction are widely researched vessel-targeting therapies (Figure 4). For example, an early study engineered *L. lactis* NZ9000 to express recombinant endostatin, a powerful inhibitor of endogenous angiogenesis. The administration of this engineered strain led to an increased mean survival time in a DMH-induced CRC rat model [74]. In addition, endostatin was also delivered by the attenuated *S. typhimurium* mutant strain S636. When feeding constructed *S. typhimurium* to CT26 colon carcinoma-bearing mice, the colonization of strains and the expression of endostatin were observed. These resulted in an increased apoptosis level and the suppression of tumor angiogenesis and ultimately caused the inhibition of tumor growth [75]. Another antiangiogenic protein, tumstatin, also a type of inhibitor of angiogenesis, is similar to endostatin. Both are from the noncollagenous domain fragments of collagen molecules [76]. Wei et al. developed a Tumstatin delivery system using *Bifidobacterium longum*, which is nonpathogenic and anaerobic, as a vector. The pBBADs promoter was employed to control the expression of Tumstatin on this vector. This Tumstatin delivery system significantly reduced the microvessel density of the tumor, induced the apoptosis of vascular endothelial cells, and inhibited tumor growth in CT26 tumor-bearing mice [77]. Recently, it was reported that bacteria that colonize tumors triggered local inflammation and induced tumor thrombosis [78,79]. Based on this, Qin et al. constructed an engineered strain therapeutic platform, AIB@ClyA, using *E. coli* K-12 strains. After the engineered strains were administered to mice bearing CT26 tumors, they first accumulated and proliferated within the tumors; this led to intratumoral thrombosis and nutrient deprivation. Next, the ClyA protein expression caused membrane perforation and further exacerbated tumor thrombosis. Based on the above, the AIB@ClyA strategy resulted in a 79% tumor proliferation reduction and decreased tumor metastasis [61]. However, it did not seem to achieve the desired therapeutic effect via anti-angiogenesis and the destruction of tumor blood vessels due to the resistance mechanisms driving tumor regeneration [80]. An emerging concept is to promote the “normalization” of abnormal tumor vasculature, which more effectively delivers drugs and oxygen to cancer cells and thus improves the efficacy of radiotherapy and chemotherapy [80,81]. Therefore, these studies illustrate the complexity and flexibility of tumor treatment based on targeting the vasculature.

#### 3.1.4. RNA Interference (RNAi) Molecules

**Figure 5:**
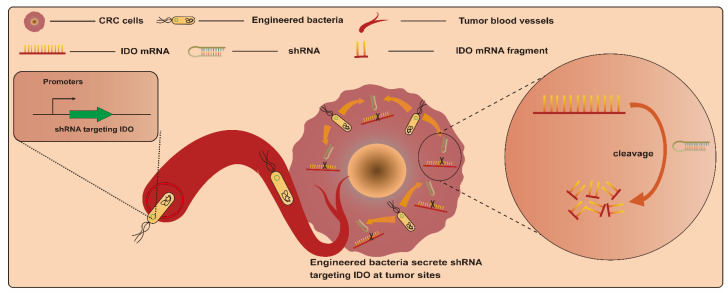
Engineered bacteria harboring a shRNA plasmid targeting indoleamine 2,3-dioxygenase (IDO) can activate the CTNNB1 pathway and induce colorectal carcinogenesis, with the shRNA employing bacteria as a vector to transport therapeutic RNAi target cells to silence specific mRNAs.

RNAi is an efficient gene-silencing mechanism, and the expression of various genes of interest can be effectively suppressed via RNAi. This makes it a potential tumor gene therapy tool via the silencing of particular genes associated with cancer induction. However, RNAi-mediated therapy in vivo faces the challenge of insufficient targeting [82,83]. Based on this, bacteria-mediated RNAi therapy may provide a good alternative strategy due to the excellent tumor-targeting properties of bacteria. Bacteria-mediated RNAi therapy involves employing bacteria as a vector to transport therapeutic RNAi effectors, such as short hairpin RNA (shRNA), into target cells to silence specific mRNA [84]. An earlier study engineered *E. coli* BL21DE3 strains to produce a shRNA. This interfering shRNA expressed by engineered strains in vitro and in vivo significantly induced the silencing of the cancer gene CTNNB1 (catenin beta 1), whose overexpression activates the CTNNB1 pathway and induces colorectal carcinogenesis [85] (Figure 5). This is the first confirmation of the feasibility of targeting genes in mammalian cells with engineered bacteria-producing interfering RNAs. After that, Guo et al. employed attenuated *S. typhimurium* rather than *E. coli* as a vector to deliver shRNAs. This strategy led to a significant reduction in CTNNB1 gene expression in vitro and in vivo as well. In addition, engineered-strain-mediated CTNNB1 knockdown remarkably inhibited tumor growth and reduced the number and size of polyps, respectively, in mice with SW480 xenograft tumors and APC^Min^ mice [78]. Another example involves the delivery of shRNA by *S. typhimurium* targeting the inhibin alpha subunit (sh-INHA). INHA protein is overexpressed in CRC cells and tissues compared with normal counterparts, which may contribute to the development of CRC. As a result, *S. typhimurium*/sh-INHA resulted in the downregulation of INHA expression and the activation of caspase, as well as the downregulation of the expression of B-cell lymphoma 2 (Bcl-2) and B-cell lymphoma-extra-large (Bcl-xL) in CRC cells [79]. The administration of *S. typhimurium*/sh-INHA suppressed tumor growth and extended the survival of mice bearing CT26 tumors [79]. Notably, one study focused on modulating immune responses for CRC treatment via RNAi. In this study, an engineered attenuated *S. typhimurium* strain armed with an shRNA plasmid targeting indoleamine 2,3-dioxygenase (shIDO-ST) was developed. CRC cells were immunosuppressedby the overexpression of indoleamine 2,3-dioxygenase (IDO). The administration of shIDO-ST downregulated the expression of IDO in the TME and activated innate immunity, finally resulting in an attenuation of tumor growth in a murine CT26 and MC38 tumor model [86].

#### 3.1.5. Immunoregulatory Factors

**Figure 6:**
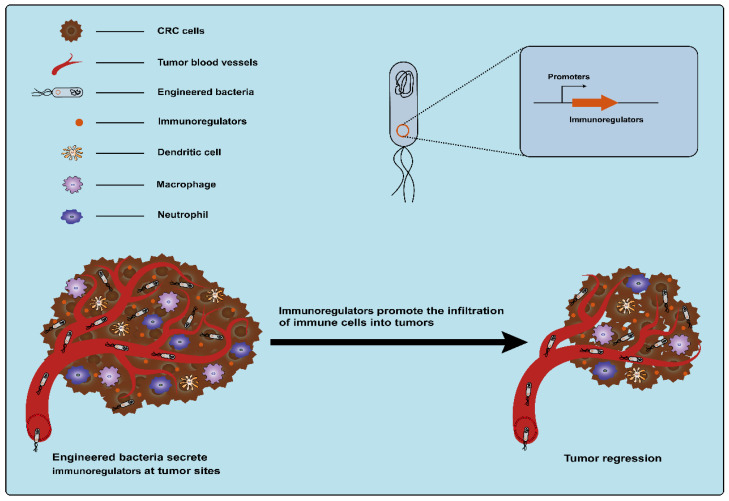
Engineered bacteria harboring immunoregulators can modulate innate and adaptive immune responses and promote the infiltration of immune cells into tumors.

Cytokines, secreted by various types of cells, are classic immunomodulators. Immune cells can be induced by cytokines to clear tumors by modulating innate and adaptive immune responses (Figure 6) [17]. However, monotherapy using cytokines is often accompanied by severe dose-limiting toxicity and the therapeutic outcome is affected by the route of administration, schedule, and strategies [87,88]. The precise delivery of cytokines to tumor sites using bacteria as a vehicle can reduce off-target effects and systemic toxicity. This may be an alternative strategy for using cytokines to treat cancer with great potential. The use of engineered bacteria-carrying cytokines for the treatment of CRC has been extensively explored. As early as 1996, an engineered attenuated *S. typhimurium* strain that enabled the production of the bioactive cytokine IL-2 was developed, and the administration of the engineered strain significantly reduced liver metastasis from MC38 colon adenocarcinoma in a mouse model of liver metastasis [89]. After that, one study also used attenuated *S. typhimurium* to deliver another human cytokine, LIGHT, which is homologous to the TNF family cytokine lymphotoxin and can promote tumor rejection. The engineered *S. typhimurium* expressing LIGHT significantly inhibited the growth of tumors and the dissemination of lung metastatic tumors in CT26 tumor-bearing mice [90]. Another example is the modification of attenuated *S. typhimurium* to deliver the cytokine IL-18, an interleukin family member that enhances immune cell activity. These engineered *S. typhimurium* strains expressing IL-18 (but not control strains) inhibited the growth of tumors in mice with CT26 tumor cells, without apparent toxicity within normal tissues [91]. Subsequently, other cytokines, such as Fas ligand (FasL) and CCL21, were also delivered to tumors by an attenuated strain of *S.typhimurium*. Among these factors, FasL, a cytokine belonging to the tumor necrosis factor (TNF) family, exhibits potential antitumor properties, such as facilitating tumor rejection and stimulating the proliferation of CD8+ T-cells [92]. CCL21 belongs to the chemokine family. The activation of CCL21 can effectively induce an immune response to eliminate tumors [93]. It was observed that the systemic delivery of both of these engineered *S.typhimurium* expressing these cytokines significantly suppresses tumor growth in BALB/c mice bearing CT26 colon carcinoma tumors with minimal toxicity [92,94]. In addition, Zheng et al. engineered an attenuated *S. typhimurium* strain to produce flagellin B (FlaB) derived from *Vibrio vulnificus*, which can also serve as an immunomodulatory agent. Lipopolysaccharide (LPS) from *S. typhimurium* and FlaB expressed by *S. typhimurium* can activate the TLR4 and TLR5 pathways, respectively, which results in the massive infiltration of immune cells into the TME and promotes the secretion of proinflammatory cytokines TNF-α and IL-1β to fight tumor cells [95,96]. Animal experiments showed that the engineered *S. typhimurium* expressing FlaB effectively retarded tumor growth and metastasis and promoted survival in several different murine colon cancer models [96]. Currently, therapy targeting immune checkpoints is an important means of cancer immunotherapy. However, there are also toxic side effects caused by systemic administration. Therefore, Gurbatri et al. engineered a probiotic EcN system for the release of nanobodies targeting programmed cell death–ligand 1 (PD-L1) and cytotoxic T-lymphocyte-associated protein-4 (CTLA-4). In this system, a synchronized lysis circuit (SLC) was employed to control the lysis of the chassis by sensing the EcN density within the tumor, which ensured that the bacterial population was enriched at the tumor site. Compared to conventional clinical therapy, this strategy induced an enhanced therapeutic response, including relative increases in activated T cells and systemic memory T cell populations, and eventually led to tumor regression in syngeneic CT26 murine models [97]. Another study reported that engineered attenuated *S. typhimurium* with a shRNA plasmid was used for the downregulation of the expression of the immune checkpoint protein IDO. This strategy activated innate immunity and inhibited CRC growth in mice and was superior to IDO inhibitor epacadostat and anti-PD1 antibody [94].

### 3.2. Engineered Bacteria Mediate CRC Treatment via Outer Membrane Vesicles

Several Gram-negative bacteria continuously release vesicles called outer membrane vesicles (OMVs) to the outside. The components of secreted OMVs include outer membrane proteins, lipopolysaccharides, phospholipids, and some periplasm [57]. OMVs are involved in both internal and external bacterial stress responses and play an important role in bacterial growth and survival [98]. OMVs possess nano-size and immunomodulatory properties [99], which may make them potential cancer immunotherapeutic agents. Kim et al. reported that lipidA acyltransferase-deficient *E. coli* (∆msbB)-derived ΔmsbB OMVs were used as cancer immunotherapeutic agents. In CRC mice, ΔmsbB OMVs targeted tumor tissues via enhanced permeability and retention, and the trypsin-sensitive surface proteins on OMVs then induced the production of interferon-γ to activate antitumor immune responses. This is an exciting attempt to apply OMVs to the treatment of cancer [100]. Through a bacterial sorting mechanism, several proteins were carried by OMVs, leading to the selective localization of proteins, such as the pore-forming toxin ClyA, from the periplasmic and outer membranes. A study indicates that C-terminal ClyA fusion heterologous proteins can be displayed on the outer surface of *E. coli* and the OMV surface and still retain activity. Interestingly, the hemolytic activity of ClyA is lost in this process. These results show that bacteria-derived-OMVs can be engineered as potential drug-delivery vehicles. In one example, a hyper-vesiculating EcN (ΔECHy) producing recombinant OMVs packaged with ClyA-hyaluronidase (Hy) was developed for tumor-targeting therapy [19]. ΔECHy can preferentially locate within tumors, followed by the release of OMVs with therapeutic molecules in situ. The recombinant OMVs easily reach deep into the tumor to induce tumor stromal changes due to their nanoscale size. Significantly, in MC38 tumor mice, ΔECHy treatment resulted in reductions in the hyaluronic acid synthesis and smooth muscle actin of tumor tissues. The combination therapy of ΔECHy+PDL1 antibody significantly suppressed tumor growth and improved survival by enhancing the efficacy of therapeutic antibodies and facilitating immune cell infiltration [19]. Another study involved the development of an oral tumor vaccine using engineered bacteria-producing OMVs [21]. Specifically, engineered *E. coli* Top10 was constructed to express a designated tumor antigen (Ag) along with an Fc fragment from mouse immunoglobulin G (Ag-mFc), both regulated by a monosaccharide-inducible promoter. Ag-mFc was fused with the protein ClyA, which was located on the surfaces of OMVs released by the engineered bacteria. In mice, OMVs from engineered bacteria can cross the intestinal epithelium into the lamina propria to stimulate dendritic cell maturation after the oral administration of engineered bacteria. The results showed that engineered bacteria activated the tumor antigen-specific immune response and inhibited the tumor growth in MC38 colon cancer mice [21]. In summary, in contrast to delivering therapeutic proteins directly using engineered bacteria, delivery via OMVs may be even more advantageous. This is because OMVs have immunogenic properties that contribute to reducing the burden of the administration of bacteria, and their nano-size gives OMVs an excellent tumor penetration ability. However, in future studies, the safety can be meticulously evaluated due to their bacterial origin..

### 3.3. CRC Treatment via Metabolic Engineering of Engineered Bacteria

Instead of delivering therapeutic proteins by constructing recombinant plasmids, another strategy is to reprogram bacteria via the design of metabolic pathways for synthesizing therapeutic molecules [22,101]. For instance, an EcN strain was developed to generate an engineered strain, EcN-BUT, that can synthesize biobutyrate from glucose. In this strain, the heterologous gene clusters phaA, hbd, crt, and ter were integrated into the EcN genome to construct a metabolic pathway from glucose to butyryl-CoA. Next, overexpressed endogenous atoDA converts butyryl-CoA into biobutyrate. In addition, the endogenous aceEF was added to enhance the expression level of acetyl-CoA, and some competing reaction processes that may affect acetyl-CoA synthesis also were blocked. Finally, EcN-BUT synthesized biobutyrate reaching 20 mM. In vitro, the treatment of HT29 CRC cells with produced biobutyrate led to cell cycle arrest in the G1 phase and induced apoptosis. In HT29 tumor-bearing mice, the administration of EcN-BUT showed tumor-specific colonization and a 70% reduction in tumor volume [102]. Another strategy targeted the pathway for the synthesis of ammonia to L-arginine. In this study, EcN was also used as a modified therapeutic vector. First, the arginine repressor gene (ArgR) encoding the negative regulator for l-arginine biosynthesis was deleted from the EcN genome. Next, N-acetyl glutamate synthase (ArgA) was replaced by ArgA^fbr^, which works without being affected by high levels of L-arginine. In MC38-tumor-bearing mice, this metabolically engineered EcN strain was able to colonize tumors and continuously convert ammonia, which accumulates at tumor sites as a metabolic waste product, into arginine. An increased arginine concentration enhances anti-tumor T-cell responses. This strain combined with programmed death-ligand 1 (PD-L1)-blocking antibodies resulted in the complete eradication of MC38 tumors in 74% of mice [103]. This metabolic engineering strategy, based on bacterial genome modification, enables the synthesis of other therapeutic molecules in addition to proteins and removes limitations on the type of therapeutic substance delivered. Notably, the reconstruction of the microbial metabolic pathways needs to consider whether it affects microbial growth and whether the concentration of the target product reaches a functional level.

## 4. Probiotics and Gut Microbiota

Changes in the gut microbiota can promote colorectal carcinogenesis. Nakatsu et al. analyzed the microbial community in the human colorectal mucosae at different stages of colorectal tumor development. The results revealed significant mucosal microbiota alterations throughout the various stages of colorectal carcinogenesis. The microbial communities in the tumor and tumor-adjacent mucosae also exhibited inconsistency [104]. Subsequent investigation revealed that fecal samples from CRC patients can induce tumorigenesis in germ-free mice, indicating that microbial dysbiosis contributes to the initiation and progression of CRC [105]. Specific bacteria from gut microbiota with dysregulation play a dominant role in promoting carcinogenesis. *F. nucleatum* abundantly accumulates in the tumor tissues of late-stage CRC patients [106]. The bacterial adhesin FadA, secreted by Fn, facilitates bacterial adherence and the invasion of colonic epithelial cells, resulting in the activation of cancer-related signaling pathways such as β-catenin and Wnt, ultimately inducing colonic tissue carcinogenesis [107]. *Enterotoxigenic Bacteroides fragilis* (ETBF), an additional CRC-associated pathogenic bacterium, can colonize the intestinal mucosa and continuously release the *Bacteroides fragilis* toxin (BFT) [108]. The accumulated BFT stimulates the development of colonic precancerous lesions and carcinogenesis, suggesting that it has the potential to serve as a biomarker for the early detection of CRC [109]. *E. coli* harboring the pathogenic pks gene island (pks^+^ *E. coli*) are frequently present in the guts of healthy individuals as commensal bacteria with conditional pathogenic potential. The abundance of pks^+^ *E. coli* in the colonic mucosa of CRC patients is significantly higher compared with that of healthy individuals [110,111]. The pks gene island enables pks^+^ *E. coli* to produce and secrete the genotoxic colibactin, interacting with host cell DNA to cause DNA strand crosslinking, breakage, and the induction of specific T-base substitutions, deletions, and insertional mutations [112].

## 5. Application of Probiotics in Combination Therapy

Probiotics have shown promising therapeutic effects on CRC, yet relying solely on single-probiotic therapy remains impractical for comprehensive tumor treatment. Excellent chemotherapy drugs (5-Fu) are widely used in clinical applications [113]. Nonetheless, certain studies have utilized probiotics as a complement to drugs to achieve superior outcomes. For example, as an approved antitumor agent, celecoxib exhibited a preventive effect on CRC. In contrast, the combination therapy of *L. rhamnosus* GG and celecoxib resulted in a greater reduction (56%) in the number of aberrant crypt foci than either treatment alone (*L. rhamnosus* GG: 27%; celecoxib: 40%) in DMH-induced CRC Sprague-Dawley rats [114]. Another study involved the combined administration of capecitabine and probiotics [*L. plantarum*, *L. casei*, *Streptococcus faecalis*, and *B. brevis*] in Wistar-Lewis rats with DMH-induced CRC. It was found that the tumor burden in the combination treatment group (1.25) was lower than in the capecitabine group and probiotics group (1.81 and 3.9) [115].

Cancer monotherapies are insufficient to produce satisfactory results due to tumor heterogeneity, which is associated with acquired drug resistance [82,116]. Engineered probiotic-based combinatorial therapies can be individually designed based on multiple characteristics of cancer to deal with dilemma at hand. For instance, the efficacy of anti-PD-L1 antibody-mediated immunotherapy is reduced due to lower L-arginine levels in solid tumors [117]. To solve this problem, probiotic EcN was developed to be L-Arg bacteria, which can colonize the tumor site and continuously synthesize L-arginine. Notably, the substrate used by L-Arg bacteria is ammonia, the metabolic waste product in tumors, which represents a strategy that turns waste into treasure. The results indicated that in MC38 colon cancer mice, the combination therapy of L-Arg bacteria with anti-PD-L1 antibodies increased the efficacy of alone anti-PD-L1 therapy by nearly 30% [103].

Additionally, some in vitro studies also proved the advantage of combination therapy [118,119]. For example, the combination administration of 5-Fu and an *L. plantarum* culture supernatant promoted apoptosis in the chemotherapy-resistant colon cancer cells HT-29 and HCT-116 via enhancing cellular hypersensitivity to 5-Fu [120]. Although the direct anticancer effects of probiotics are generally weaker than chemotherapy drugs, probiotics offer a variety of advantages such as regulating gut microbiota and alleviating chemotherapy side effects. Combining them with chemical drugs for CRC treatment may possess significant therapeutic benefits in clinical applications because they complement each other’s strengths and weaknesses.

## 6. Conclusions and Future Perspectives

To date, considerable progress in the preclinic has been made in CRC therapy based on engineered bacteria. However, there are no reports of clinical trials, and several questions have yet to be addressed before bacteria can be applied in the clinic. Several reviews have discussed the limitations of probiotic-mediated CRC therapy [8], including systemic infections, deleterious metabolic activities, excessive immune stimulation in susceptible individuals, and resistance gene transfer; this study mainly focuses on the mechanism of probiotic-mediated CRC therapy [15] and the delivery of therapeutic payloads using engineered bacteria for improving the therapeutic efficacy of CRC. Nonetheless, many issues need to be addressed before engineered bacteria can be used clinically. Although the toxicity of bacteria can be attenuated with gene engineering, any residual toxicity may be fatal for advanced patients with poor immunity. In addition, the use of bacteria as anticancer agents for patients with clinical cancer and their toxicity and side effects require rigorous verification by different clinical trials and long-term follow-up. Similarly, the use of low doses of bacteria to exert excellent therapeutic effects should be the focus of attention.

The genetic instability of engineered bacteria arises from the potential loss and mutation of the recombinant plasmid carried within the bacterial host over successive generations, particularly in the absence of antibiotic pressure. This instability poses various risks, including treatment failure and increased infection severity. One potential solution is to modify the bacterial genome directly to achieve the desired outcome. However, this approach necessitates careful consideration of how genomic alterations might impact bacterial survival. Achieving a stable inheritance of engineered bacteria within the host remains a significant challenge, indicating the need for further research and development in this area.

Regarding the biosafety of bacterial administration, bacteria are living organisms, and they may mutate in patients and proliferate in the human body. The occurrence of septicemia will be another disaster for patients. As such, the clinical development of bacterial therapy faces substantial hurdles due mainly to the potential adverse effects of infection. Furthermore, unlike other small molecules or clinical agents, live bacteria cannot be sterilized by filtration or heating. The kind of liquid used for bacterial dilution, the conditions to be used, and their effect on bacterial activity are problems in clinical translation as well. In addition, significant systemic infection with bacteria and the risk of toxicity by injection exert a psychological burden on patients, which results in decreased treatment compliance by patients. Therefore, the oral administration of bacteria without affecting efficacy may be an optimal approach.

Regarding bacterial clearance in vivo, after the tumor has subsided, suitable antibiotics can be used to kill bacteria in the host. However, the amount of antibiotics, the resistance of the host, and the destruction of balance in the host’s gut microbiota are new problems that need to be solved. Moreover, antibiotic abuse presents a significant global challenge. The excessive usage of antibiotics hastens the proliferation of drug-resistant bacteria.

## Figures and Tables

**Table 1:** Clinical trials using probiotics for the prevention and treatment of CRC.

Probiotic Strain/Symbiotic Intervention	Types of Patients/Sample Size	Duration	Key Outcomes	Refs.
Wheat bran/*Lactobacillus casei*/wheat bran + *Lactobacillus casei*/no treatment	Patients who have had at least 2 colorectal tumors removed (*n* = 380)	4 years	Wheat bran alone: multivariate-adjusted OR for the occurrence of tumors was 1.31; *L. casei* group: multivariate-adjusted OR for the occurrence of tumors was 0.76; Wheat bran + *Lactobacillus casei* group: no notable synergistic effects observed	[45]
Symbiotic (*Lactobacillus rhamnosus* GG, *Bifidobacterium lactis Bb12* + oligofructose-enriched inulin)	43 polypectomized patients and 37 CRC patients (*n* = 80)	12 weeks	Polypectomies patients: increased *Bifidobacterium* and *Lactobacillus* and decreased *Clostridium perfringens* in fecal flora; reduced exposure to genotoxins; suppressed IL-2 increase CRC patients: increased the number of *Bifidobacterium*; promoted IFN-γ production	[46]
Probiotics containing six viable microorganisms of *Lactobacillus* and *Bifidobacteria* strains	Patients with CRC at 4 weeks after surgery (*n* = 52)	6 months	Probiotics group: reduced the levels of serum pro-inflammatory cytokines TNF-α, IL-10, IL-12, IL-17A, IL-17C, and IL-22 below their respective baseline levels; the level of IL-6 decreased to 1.44 ± 1.39 pg/mL. Placebo group: increased the levels of TNF-α, IL-12, IL-17C, IL-22, IL-10, and IL-17A; reduced the level of IL-6 to 0.91 ± 0.49 pg/mL	[47]
Dietary symbiotic Simbioflora	Patients with CRC who were preparing for colorectal resection surgery (*n* = 73)	7 days	Synbiotic group: Postoperative infectious complications (2.8%); mean antibiotic usage time (1.42 days); length of hospital stays (3.0 days); no morbidity Placebo group: Postoperative infectious complications (18.9%); mean antibiotic usage time (3.74 days); length of hospital stays (4.0 days); three deaths	[48]

Note: IL-2: Interleukin-2; IFN-γ: Interferon-gamma; TNF-α: tumor necrosis factor-alpha; IL-6: Interleukin-6; IL-10: Interleukin-10; IL-12: Interleukin-12; IL-17A: Interleukin-17A; IL-17C: Interleukin-17C; IL-22: Interleukin-22.
